# Indirect effects of contextual factors on patients’ consultations with healthcare professionals about health information found online

**DOI:** 10.1186/s12913-016-1713-y

**Published:** 2016-08-30

**Authors:** Younsook Yeo

**Affiliations:** Department of Social Work, St. Cloud State University, 720 Fourth Avenue South, St. Cloud, MN 56301-4498 USA

**Keywords:** Health empowerment, Psychological empowerment, E-health

## Abstract

**Background:**

E-health users are encouraged to consult healthcare professionals about the health information they found online because it facilitates e-health users to participate in an informed decision-making process with healthcare professionals on treatment options. However, few studies have examined the path of how e-health users consult healthcare professionals about the health information. Using psychological empowerment, which claims that empowering individuals requires understanding contextual factors that interact with the individuals’ intrapsychic factors, this study tested a hypothesis: the contextual factors play an indirect role between patients’ perceived poor health and their consultations with healthcare professionals about the health information found online, holding predisposing factors constant.

**Methods:**

The data were collected from the Health Information National Trends Survey and used a subsample of e-health users who used healthcare services during the past year. The subsample (*N* = 2,297) was analyzed using structural equation modeling (SEM).

**Results:**

The SEM analysis supported the hypothesized indirect model. Meanwhile, patients with low socioeconomic statuses tended to score high in the outcome measurement of the contextual factors; however, they tended not to consult professionals.

**Conclusions:**

It is important to acknowledge contextual factors, which encompass communication and relational aspects as well as the process and outcomes of treatments, when empowering e-health users to use e-health tools meaningfully and become empowered in caring for their own health. Particularly, those with low income and education levels were the less powered or powerless patients: they tended not to be competent in having a voice and discussing the health information that they found online with professionals.

**Electronic supplementary material:**

The online version of this article (doi:10.1186/s12913-016-1713-y) contains supplementary material, which is available to authorized users.

## Background

Autonomous searches for healthcare information are important in health empowerment as they help individuals acquire the knowledge and skills necessary to care for their own health and participate in an informed decision-making process with healthcare professionals on treatment options [1–3]. Internet-based health information and communication (i.e. e-health [4, 5]) is considered to be a medium for health empowerment [5–8]. The US government articulates individuals’ use of e-health as one of the key themes in its strategic plans, *Healthy People 2010* [9] and *Healthy People 2020* [1], which delineate a set of goals and objectives designed to guide US health promotion and disease prevention efforts. However, there exist rising concerns about the quality of health information on the Internet: some of the information is inaccurate, complex or even fraudulent [10–14]. Some online searchers alter their treatment regimens or do not adhere to the treatment recommended by their physicians [15, 16]. Hence, e-health users are encouraged to consult healthcare professionals about the health information that they find online in order to achieve the ultimate goals of health empowerment. However, research has shown that the rates of e-health users who discuss the health information with their healthcare professionals are low [17–19]. For example, approximately 59 % of the US population self-diagnosed medical conditions on the Internet in 2013; only 53 % of these individuals talked to their physicians about the information [17].

The contexts that affect how individual manage their health on a daily basis are multilayered, including health policies and systems and health service providers [20–22], suggesting that the process of health empowerment can be facilitated by contextual factors although health empowerment can also be individually achieved. Previous studies have focused on how individuals use e-health tools for health [23], whether they consult their healthcare providers about the information that they found online [18, 19, 23], and what individual characteristics hinder e-health users from consulting with their healthcare professionals about online health information [24]. However, few studies have examined the path of how e-health users consult healthcare professionals about the health information that they found. This study aimed to examine this path in relationship to patient empowerment. A systematic review of the literature acknowledged that not only patients’ knowledge but also the influence of a power imbalance between patient and doctor affect the shared decision-making [25]. Hence, the strength of the present study includes its empirical test of the path using a theoretical framework of psychological empowerment [22], which acknowledges the effects of the contexts on the individuals’ perceptions and behaviors and the influences of the power differential between patients and healthcare professionals.

### Patient empowerment

The empowerment of patients is gaining importance in healthcare settings [26–28]. However, the best way to define and measure patient empowerment is still unclear [26, 29–31] due mainly to the nature of empowerment, which “is theoretically inconsistent with the construct given the specific demands and characteristics of different settings and life situations” ([22], p. 587). Nevertheless, empowerment can be considered both a process (i.e., empowering process) and outcome (i.e., empowered outcome) where the former refers to how individuals become empowered, while the latter refers to the consequences of those processes ([22], p. 583). A plethora of literature (e.g., [20, 25, 26, 32, 33]) depicts empowered patients as those individuals who are proactive in gaining positive health outcomes by (i) understanding their health conditions and their impact on their bodies; (ii) undertaking active participation in decision-making with their providers and making informed decisions about treatment; (iii) understanding the need to make necessary changes to their unhealthy lifestyles; (iv) actively asking questions of their healthcare providers; (v) taking responsibility for their health and actively seeking care only when necessary; and (vi) actively seeking out, evaluating, and making use of information. Patients can empower themselves to gain positive health outcomes, but a process for patient empowerment can also be “designed to help patients develop the knowledge, skills, attitudes, and degree of self-awareness necessary to affectively assume responsibility for their health-related decisions” ([21], p. 139, also see [20, 22, 25, 33–36]).

**Fig. 1:**
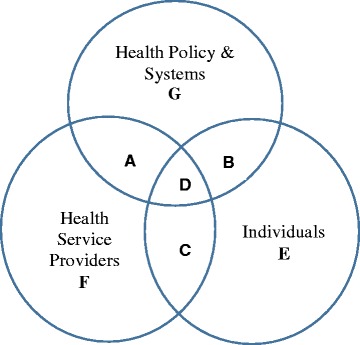
The context for health empowerment ([20], p. 31)

Individuals interact with the environment and contexts surrounding the individuals. The empowerment approach which acknowledges contexts’ (e.g., ecological, cultural, sociopolitical factors) influences on the individuals (e.g., intrapsychic factors such as cognitive, personality, and motivational aspects of control) is called psychological empowerment [22, 34–36]. This approach helps understand how individual-level characteristics including intrapsychic factors interact with what goes on in the individuals’ environment to promote or inhibit one’s mastery of control over the factors that affect one’s life [22]. According to Menon [20], the contexts which affect patients’ health comprise (i) health policies and systems, (ii) health service providers, and (iii) individuals (Fig. 1). The intertwined contexts suggest that not only health policies and systems but also health service providers affect individuals’ health empowerment activities. Bravo et al.’s [33] conceptual map of patient empowerment also illustrates potentially differential effects of the empowering process on patient empowerment depending on contextual factors (e.g., healthcare providers, healthcare system, culture, and political context).

The contextual factors in a healthcare setting may include the structure (e.g., a good quality of hospitals and healthcare professionals), process (e.g., having right things get done in the right way), and relational properties such as communication, information, and coordination [33, 36, 37]. Previous research has also showed that good health, which is one of the outcome measurement of patient empowerment, has a positive association with the outcome measurement of the contextual factors [26, 33, 38–41]. Acknowledging the importance of the psychological aspects of empowerment, health policy in the UK as well as in many other countries has been prioritizing “[patient’s] perceived value of non-health outcomes such as empowerment, a psychological outcome” ([26], p. 1) by “making hospital funding contingent upon performance against a range of quality measures, including Patient-Reported Outcome Measures” ([26], p. 2) in order to improve the quality of care from the patient’s perspective [26–28]. According to Anderson [37], this approach empowers patients since it views patients as major stake-holders in healthcare in that they are individuals who “have a say in how health care is delivered” (p. 697).

### Health communication

Patients can also empower themselves through self-directed participation in patient organizations or community activism or through self-education facilitated by the Internet [29]. The use of electronic-based health information technology for health communication is called as e-health [4, 5]. Among others, the Internet is viewed as the cost-effective and secure use of information and communications technologies in support of health and health-related fields [4, 5]. Finding health information is critical to patients, in particular, those patients with chronic diseases or conditions including cancer, in regard to shared decision-making with healthcare professionals on treatment options [25]. Empirical research has also found that poor health and, thus, healthcare service use, is positively associated with searching for health information [29, 42–48]. Those patients who search for health information use the Internet, among other health communication tools, because of the ease in which it can be accessed; the anonymity it may facilitate, especially when e-health users are in need of searching for sensitive disease symptoms; and its cost-effectiveness [49–51]. It has become a critical medium in the process of patient empowerment [5, 51, 52]. The expected outcomes of patient empowerment also overlap the goals of e-health. They include self-care, seeking health information and making informed decisions on treatment, which were promoted at the first European Conference on Patient Empowerment held in Copenhagen Denmark in April 2012, in collaboration with the World Health Organization (WHO) Regional Office for Europe [32]. Nevertheless, healthcare providers should be at the center of health communication because, otherwise, e-health users may be misguided by false self-diagnoses and, thus, delay their seeking of medical care when they need it [10–14, 53, 54].

Research shows that people with poor health also tend to consult healthcare providers about the health information that they found online [29, 50, 52, 55]. However, not all patients are proactive in regard to their participation in the discussions with healthcare professionals as to the online health information for their treatment options, although they have actively searched for and found information on treatments [56, 57]. Meanwhile, Joseph-Williams and his colleagues [25] found, through a systematic review of the literature related to patient-reported barriers and facilitators, that “knowledge alone is insufficient and power is more difficult to attain” in regard to participating in discussions with healthcare providers (p. 291). In particular, socially disadvantaged and less powered populations may not actively participate in the discussions with healthcare providers [35, 37].

Previous studies have documented factors that hinder e-health users from taking the health information that they found online to their healthcare providers. These studies have focused on patient-related factors, including inadequate knowledge and skills in managing disease symptoms and treatment as well as other psychosocial influences (e.g., low motivation, low self-esteem, anxiety about disease symptoms) [24, 49, 58]. However, a lack of studies exists that examine the effects of the contextual factors (e.g., a good quality of hospitals and healthcare professionals, having right things get done in the right way, and relational properties) in patient empowerment on e-health users’ consultations with healthcare professionals about the health information they found online. Hence, to fill the gap in knowledge, this study tested a hypothesis that the contexts in patient empowerment (mediator [M]) will play an indirect role between poor health (IV) and consultations with healthcare providers about the health information found online (DV) among e-health users (Fig. 3).

## Methods

### Data and sample

To test the postulated hypothesis, this study used the Health Information National Trends Survey (HINTS), which has been administered every few years since 2003 by the U.S. National Cancer Institutes. Its ultimate goal is to learn the patterns of how adults find, understand, and use health information. It plans to achieve this goal by collecting data on health-related communications, patterns of communication with doctors, and behaviors related to the Internet, health services, and health information technology. It is one of the most comprehensive national-level datasets for these topics in existence [59].

The data collection procedures for this dataset encompassed a complex, multistage sampling designed to represent the civilian, non-institutionalized population of the United States. It encompassed samples from both a telephone random digit dialing sample of phone numbers and the mail through a sample of addresses [60]. For this study, the researcher used HINTS data collected between January 2008 and May 2008 (*N* = 7,674). A subset of the sample for this study contained those (≥18) who (1) went to the Internet first to look for information about health and medical topics; (2) used healthcare services during the past 12 months; and (3) gave valid data. The final unweighted sample consists of 2,297 respondents.

### Measures

A measurement of patient empowerment has many constructs, but is not well constructed, leaving “[uncertainty] about the best way to define and measure it” ([30], p. 1, also see [8, 29]). Moreover, Zimmerman [22] acknowledged that it is unlikely that an empowerment measurement “would [universally] fit all (or most) persons” and “would [globally] fit all (or most) contexts” (p. 587). The measurement of psychological empowerment, however, may measure the consequences of the empowerment process, including an examination of “the effects of interventions designed to empower participants [and] empowering processes and mechanisms” ([22], p. 585). The latent concept of empowerment can be “potentially measurable” using the manifested perceptions and behaviors as to empowering process as well as empowered outcomes ([33], p. 1).

Literature revealed that an outcome measurement of the quality of healthcare services encompasses the structural properties such as facilities and healthcare professionals, the process and outcome of the treatment, and relational properties such as communications, information, and coordination [39–41, 61], which overlap with the contextual factors in patient empowerment [22, 25–28, 34, 35]. The patient empowerment measurements, which asked the patients’ perceptions of the quality of healthcare services, included Small et al.’s [62] and Bulsara et al.’s [63] patient empowerment scales. The former used one question to measure patients’ trust in their doctors and seven items to measure the patients’ perceptions of their doctors’ interpersonal care skills (e.g., listening to the patients, involving the patients in decisions, treating the patients with care, and taking the patients’ problems seriously) while the latter used a single item to ask about patients’ perceptions of their healthcare professionals’ willingness to include them in the decision-making process for treatment. Patients’ assessments of the quality of healthcare services may well capture the patients’ global perceptions of how the healthcare services (i.e., contextual factors) were facilitated to empower the patients to talk with their healthcare providers about the health information they found online like a measurement of self-assessed health. A measurement which asks individuals about universal health is considered to be as reliable and valid as biological measures, such as physical and laboratory examinations [64]. Hence, the outcome measurement of the contextual factors in psychological empowerment was operationalized as “Overall, how would you rate the quality of health care you received in the last 12 months?” This question was measured on a 5-point Likert scale with higher numbers representing greater empowerment outcomes.

The patients’ self-assessed general health (from 1 = excellent to 5 = poor) and psychological distress were used to represent the health latent variable. Psychological distress contains six constructs (i.e., sad, nervous, restless, hopeless, taxing, and worthless), which the patients could have experienced over the past 30 days. Each item was measured on the 5-point scale (from 1 = all of the time to 4 = none of the time) and the scores were reversed for this study. The summation of these constructs ranged from 1 to 24. Hence, higher scores indicated poor physical and mental health.

The patients’ predisposing factors to be controlled were cancer history (binary) and socio-demographics. Socio-demographics included age (18–34, 35–54, 55–74, or 75+), gender (male vs. female), race and ethnicity (i.e., non-Hispanic whites, Hispanics, blacks, or Other), marital status (not married vs. married), education (i.e., high school, some college, or college+), job (unemployed vs. employed), household income (<20 K, <35 K, <50 K, <75 K, 75 K+), and U.S.-born (yes vs. no). Residential area (<250,000 for urban vs. ≥250,000 for rural) was descriptively analyzed, but excluded in the structural equation modeling (SEM) due to its non-significant impact on any paths of the model.

The binary dependent variable (i.e., a consultation with healthcare providers about health information found from the Internet) was operationalized as “In the past 12 months, have you talked to a doctor, nurse, or other health professional about any kind of health information you have gotten from the Internet?” This variable is only available from HINTS 2, HINTS 3, and HINTS 4 Cycle 1, for which the data were collected in 2005, 2008, and 2011, respectively [65]. This study used HINTS 3 (*N* = 7,674) because the total sample size was almost double that in HINTS 4 Cycle 1 (*N* = 3,959), which reduces the sampling error and produces better estimates of the U.S. population given, in particular, the present study subsample as described in the Data and Sample section.

### Data analysis

There were two steps to the analyses. First, the study variables’ univariate analysis for the sample characteristics and bivariate relationships of the study variables with respect to whether taking the health information found online to healthcare professionals were conducted in the SAS statistical software version 9.2 (see Additional file 1). In order to account for HINTS’s survey design and complex multistage sampling design, all of the data were weighted in the descriptive analyses, using post-stratification weights with Jackknife repeated replication methods. These methods allowed for accurate estimates of the variance for the full sample, which, in turn, affected the standard errors, p-values, and confidence levels in the inferential statistical analysis with HINTS [59]. The univariate distribution and bivariate relationships with respect to consultations with healthcare professionals were examined using Rao-Scott chi-square tests for the categorical data and t-values for the summated psychological distress, which were regressed upon the dependent variable. The chi-square values and t-values were calculated using the PROC SURVEY-procedures.

Next, to test the hypothesis for the proposed indirect paths, SEM in Mplus was used, not only because it allowed us to test complex paths and multiple regressions for the model, but also because it is a comprehensive means for assessing and modifying theoretical models, which led to further theory development [66]. This study used a robust maximum likelihood estimator (i.e., MLR option in Mplus) using Monte Carlo integration with 500 integration points. This method is robust for categorical data that has violated the underlying normality assumption because it produced robust standard errors [67].

To see whether an indirect effect exists in the proposed model, the following four steps guided by Baron and Kenny [68] were used: Confirm (1) IV was significantly correlated with DV (= c); (2) IV was significantly correlated with M (= a); (3) M affected DV while controlling for IV (= b); and (4) the total effect (= *c*) equaled the summation of the direct effect (= *c*′) and indirect effect (a*b).

## Results

### Sample characteristics

Table S1 in Additional file 1 showed that younger adults tended to comprise the sample of those individuals who used the Internet for health information and healthcare services during the past 12 months (35.3 % for 18–34, 36.4 % for 35–54, 22.7 % for 55–74, and 5.6 % for 75+). They tended to be married (63.7 %), insured (92.0 %), employed (67.7 %), U.S. citizens by birth (91.3 %), non-Hispanic whites (79.2 % for non-Hispanic whites, 7.8 % for blacks, 7.1 % for Hispanics, and 5.9 % for others), and urban dwellers (72.5 %). They also tended to be educated (21.7 % for ≤ high school, 42.2 % for some college, and 36.1 % for college+) and to have higher household income (8.6 % for <20 K, 10.2 % for <35 K, 15.2 % for <50 K, 22.9 % for <75 K, and 43.1 % for 75 K+).

Overall, the sample tended to report good health (11.2 % for excellent, 39.4 % for very good, 37.0 % for good, 10.4 % for fair, and 2.0 % for very poor) and not to report psychological distress (M = 6.03, SD = 0.12). Nearly 6.1 % of the sample had been diagnosed with cancer at some point in time. Approximately 8 % of the sample reported ‘fair’ or ‘very poor’ as their perception of their empowerment. Those individuals who talked to healthcare professionals about the health information that they found online were 35.3 % of the sample.

### The bivariate relationship of the study variables

Healthcare consumers who took the health information that they found online to healthcare professionals tended to report poor health (*p* < 0.05) and perceive being empowered (*p* < 0.1). They tended to have higher household incomes (*p* < 0.1). The highest rates of consultation with professionals were observed among those individuals who self-identified as blacks (45.5 %), followed by non-Hispanic whites (35.0 %), ‘others’ (32.0 %), and Hispanics (29.3 %). Non-significant differences were observed in age, gender, marital status, insurance, job, education, residential area, and U.S. citizenship by birth.

### Indirect effects of perceptions of being empowered

**Fig. 2:**
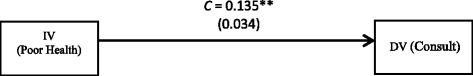
A direct effect model: relationship between perceived poor health (IV) and consultation with healthcare providers about health information found online (DV) among e-health users. †*p* ≤ 0.1. *p ≤ 0.05. **p ≤ 0.01. ***p ≤ 0.001

**Fig. 3:**
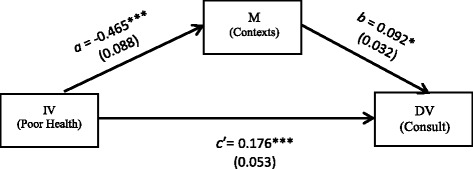
An indirect effect model: an indirect role of an outcome measurement of contextual factors (M) in psychological empowerment between poor health (IV) and consultations with healthcare providers about the health information found online (DV) among e-health users. †*p* ≤ 0.1. *p ≤ 0.05. **p ≤ 0.01. ***p ≤ 0.001

The results of the direct effects model while holding the predisposing effects constant are presented in Fig. 2. The direct effect model (= ‘c’) showed a positive and significant relationship between self-reported poor health and consultation with healthcare providers about online health information (standardized regression coefficient [*β*] = 0.135, SE = 0.034, *p* = 0.002). The relationship between self-reported poor health and the outcome measurement of contextual factors (= a) was significantly and negatively associated (*β* = −0.466, SE = 0.044, *p* < 0.0001, not in Fig. 3) while holding the effects of predisposing factors constant. Then, path ‘b’ was tested and showed a significant and positive relationship between the outcome measurement of contextual factors and consultation with healthcare providers (*β* = 0.092, SE = 0.032, *p* = 0.004, see Fig. 3) while controlling for the effects of self-reported poor health and predisposing factors. Finally, the direct effect model was compared with the indirect model to test whether the total effect (= c) equaled the summation of the direct effect (= c′) and indirect effect (a*b). Plugging the standardized coefficients into the equation produced 0.133 which is approximately equal to the total effect c or 0.135, suggesting a complete mediation effect. Meanwhile, the observed negative coefficient for path ‘a’ caused an increased coefficient for path ‘c′’ as can be seen in Fig. 3. This increased coefficient due to the observed negative coefficient is called a ‘competitive mediation’ effect [69].

### Effects of the predisposing factors

Table 1 shows that patients with a low education level (*β* = −0.098, SE = 0.032, *p* < 0.001) and a low household income (*β* = −0.068, SE = 0.047, *p* < 0.05) were more likely than patients with a high education level and a high household income to report patient empowerment. However, they tended not to consult healthcare professionals about the health information that they found online: patients’ education and household income were significant and positively explained whether the patients consulted healthcare professionals about the health information that they found online (*β* = 0.103, SE = 0.028, *p* < 0.001 for education; *β* = 0.068, SE = 0.033, *p* < 0.05 for household income). Table 1 also shows that other predisposing factors that were correlated significantly and positively with the outcome measurement of patient empowerment were cancer history (*β* = 0.067, SE = 0.023, *p* < 0.01), insurance coverage status (*β* = 0.054, SE = 0.030, *p* < 0.05), and U.S. citizenship by birth (*β* = 0.073, SE = 0.030, *p* < 0.01).

**Table 1:** Effects of predisposing factors with respect to consultation with healthcare professionals and the outcome measurement of contextual factors: results from the indirect effect model in Fig. 3

Predisposing factors	Consultation with healthcare professionals				Outcome measurement of contextual factors			
	Estimate	S.E.	Est./S.E.	*P*-value	Estimate	S.E.	Est./S.E.	*P*-value
Cancer history	0.039	0.025	1.575	0.115	0.067	0.023	2.888	0.004
Age	−0.011	0.027	−0.413	0.680	0.009	0.028	0.309	0.757
Education	0.103	0.028	3.661	0.000	−0.098	0.032	−3.065	0.002
Gender	−0.030	0.025	−1.182	0.237	0.007	0.022	0.330	0.742
Marital status	0.025	0.028	0.910	0.363	−0.026	0.029	−0.907	0.365
Insurance	0.014	0.026	0.5510	0.581	0.054	0.030	1.808	0.071
Employment	−0.031	0.026	−1.175	0.240	−0.037	0.024	−1.576	0.115
Household income	0.066	0.034	1.963	0.050	−0.068	0.047	−1.433	0.152
Race/Ethnicity	0.023	0.025	0.895	0.371	−0.049	0.025	−1.955	0.051
U.S.-Born	0.006	0.026	0.227	0.821	0.073	0.030	2.430	0.015

## Discussion and conclusion

### Discussion

This study confirmed the results from previous studies: a positive relationship exists between poor health and taking the health information found online to healthcare providers [29, 50, 52], while a negative relationship exists between poor health and the outcome measurement of patient empowerment [38, 39]. The SEM analysis supported the hypothesized indirect model: perceived poor health was positively related to consulting healthcare providers about the health information found online and this relationship was mediated by the outcome measurement of patient empowerment. This result highlights that healthcare professionals seem to play a critical role in helping patients share the health information found online with professionals, which assists patients in meaningfully using e-health tools and, ultimately, being empowered.

The theoretical underpinnings of empowerment began from working with socially disadvantaged and less powered populations [35, 37, 70]. Unlike in other organizational contexts, an “asymmetry in the relations of power between [the] patients and healthcare providers” exists in doctor-patient consultations ([37], p. 703, also see [25, 71]). Patients may fail to gain influence over events in relationship to their healthcare providers [20, 22, 25]. Less powered or powerless individuals may not be competent to speak their voices. The individuals with low socioeconomic status are often marginalized and powerless in society [62, 72] as they are portrayed as “exercise[ing] little creativity or judgment in their work, have[ing] no technical expertise or authority, express[ing] themselves awkwardly, especially in public or bureaucratic setting, and not command[ing] respect” ([72], p. 56). A systematic review of the literature also acknowledged that patients need knowledge and power to participate in shared decision-making; however, knowledge alone is not sufficient and power is difficult to attain [25]. The results of this study empirically support Young [72] and Joseph-Williams et al. [25] in that the SEM analysis using the US population data revealed that those patients with low socioeconomic status scored high in the outcome measurement of the contextual factors, but they tended not to consult professionals about information that they found online, which may be because of the asymmetry in the relations of power between patients and healthcare providers [37]. They may perceive the professionals as those with absolute power over and knowledge of their health. Alternatively, they may think that their voice is ineffective in influencing healthcare providers, or they may not want their healthcare providers to feel challenged by raising questions.

### Limitations and strengths

The use of cross-sectional data revealed the inability to make any causal inferences even in SEM [66], which, therefore, warrants a longitudinal study on this topic. This study used one manifested variable to globally measure the outcome of contextual factors in patient empowerment. The measurement conceptually encompassed many constructs including relational properties such as communicational and information aspects as well as structure, process, and outcome of the treatment [61]. Not many studies measured contextual factors when studying patient empowerment. Moreover, the studies that focused in this area included information on the patients’ trust in their doctors [62] and perceptions of their doctors’ interpersonal care skills [62, 63]. Hence, a replication of this study is recommended after developing a well-constructed and valid measurement that specifically aims to measure patient empowerment, which encompasses the structural properties such as facilities and healthcare professionals, the process and outcome of the treatment and the relational properties such as communications, information and coordination. In order to develop a well-constructed and valid measurement of patient empowerment, this study argues the importance of the specification of (i) the context area where patient empowerment will be examined as in Menon’s diagram (Fig. 1) or in Bravo et al.’s [33] conceptual model and (ii) the status of patient empowerment (e.g. process, action, and/or outcome).

Even with these limitations, the results make a contribution to the knowledge development in this area, given that no studies have tested the path of e-health users’ consultations about health information in relationship to patient empowerment incorporating contextual factors. The results provide useful information to healthcare professionals in regard to preparing for communication with their powerless patients regarding the health information that they found online. Researchers can plan further research based on the results by developing a measurement of patient empowerment incorporating contextual factors and planning an intervention study aimed to empower powerless patients. Using representative U.S. population data is also a strength of this study.

### Conclusion

The indirect effects of the contexts in patient empowerment on patients’ sharing the health information that they found online were tested. The results, which showed whether the patients consulted healthcare professionals about the health information found online was explained by whether the patients perceived they were empowered through the healthcare services that they received from healthcare professionals. The results highlighted the importance of the contextual factors in patient empowerment that encompassed communication and relational aspects as well as the process and outcomes of treatments in regard to helping patients use e-health tools meaningfully and become empowered in regard to caring for their own health. Particularly, those with low income and education levels were the less powered or powerless patients: they tended not to be competent in having a voice and discussing the health information that they found online with professionals.

The psychological empowerment, which acknowledges contextual factors as well as intrapsychic factors [22, 25, 34, 72], is important in understanding the behaviors of the patients, in particular, those with low economic and education statuses, and in helping them to acquire the knowledge and skills necessary to care for their own health. The results of this study suggest that healthcare professionals should acknowledge the importance of the contextual factors in helping e-health users consult healthcare professionals about the health information found online and ultimately should empower them for the ultimate goal of health self-management. Healthcare professionals should also provide assertiveness training to the patients with low socioeconomic statuses when preparing them to engage in clear communication about their needs and knowing what questions to ask based on the information that they found online, which will help them to construct a robust mental structure and make healthy decisions [34, 73]. The empowerment process for the less powered or powerless patients should also include helping patients with emotional support and motivational comments and by validating their thoughts and opinions to increase their self-esteem and personal competence [74–76].

## Additional file

Additional file 1: Table S1. Sample characteristics and bivariate relationships of the study variables to patients’ consultations with healthcare professionals about health information found online among the e-health users (≥18). (DOCX 15 kb)
