# Supplementary material for: Indirect effects of contextual factors on patients’ consultations with healthcare professionals about health information found online
Source: BMC Health Serv Res. 2016 Aug 30;16(1):447. doi: 10.1186/s12913-016-1713-y (PMC5006620; doi:10.1186/s12913-016-1713-y)
Supplement: Additional file 1: Table S1. — Sample characteristics and bivariate relationships of the study variables to patients’ consultations with healthcare professionals about health information found online among the e-health users (≥18). (DOCX 15 kb) [file 12913_2016_1713_MOESM1_ESM.docx]

| **Table S1**  Sample characteristics and bivariate relationships of the study variables to patients’ consultations with healthcare professionals about health information found online among the e-health users (≥18). | | | | |
| --- | --- | --- | --- | --- |
|  | Sample Characteristics | Bivariate Relationship | | |
|  | Total  (N = 2297)  % /Mean | Consulted  (n = 824)  % /Mean | Not Consulted  (n = 1473)  % /Mean | *χ*^2^ /t-value |
| Age  18-34  35-54  55-74  75+ | ***  448(35.3)  795(36.4)  781(22.7)  273(5.6) | 155(33.6)  295(38.0)  283(34.8)  91(29.9) | 293(66.4)  500(62.0)  498(65.2)  182(70.1) | 3.458 |
| Gender  Male  Female | ***  812(42.4)  1485(57.6) | 304(37.9)  520(33.3) | 508(62.1)  965(66.7) | 2.052 |
| Marital status  Married  Not married | ***  1503(63.7)  794(36.3) | 548 (37.1)  276(32.1) | 955(62.9)  518(67.9) | 2.042 |
| Insurance  Insured  Uninsured | ***  2151(92.0)  146(8.0) | 776(35.1)  48(37.4) | 1375(64.9)  98(62.6) | 0.157 |
| Job  Employed  Unemployed | ***  1510(67.7)  787(32.3) | 532(34.8)  292(36.2) | 978(65.2)  495(63.8) | 0.158 |
| Education  High school  Some college  College+ | ***  367(21.7)  762(42.2)  1168(36.1) | 104(32.7)  278(35.2)  442(36.8) | 263(67.3)  484(74.8)  726(63.2) | 0.924 |
| Household income  <20K  20K-<35K  35K-<50K  50K-75K  ≥75K | ***  164(8.6)  232(10.2)  308(15.2)  525(22.9)  1068(43.1) | 50(23.7)  86(41.5)  107(37.6)  178(33.1)  403(36.4) | 114(76.3)  146(58.5)  201(62.4)  347(66.9)  665(63.6) | 8.069+ |
| Race/Ethnicity  Non-Hispanic white  Blacks  Hispanic  Others | ***  1875(79.2)  156(7.8)  122(7.1)  144(5.9) | 671(35.0)  59(45.5)  39(29.3)  55(32.0) | 1204(65.0)  97(54.5)  83(70.7)  55(68.0) | 6.135 |
| Region  Urban  Rural | ***  1680(72.5)  617 (27.5) | 608(35.3)  216(35.3) | 1072(64.7)  401(64.7) | 0.000 |
| U.S. citizenship by birth  US-born  Foreign-born | ***  2139(91.3)  158(8.7) | 769(35.2)  55(36.1) | 1370(64.8)  103(63.9) | 0.017 |
| Health Status  Excellent  Very good  Good  Fair  Very poor | ***  279(11.2)  932(39.4)  813(37.0)  219(10.4)  54(2.0) | 97(36.9)  316(32.0)  290(35.3)  93(41.6)  28(55.6) | 182(63.1)  616(68.0)  523(64.7)  126(58.4)  26(44.4) | 11.096* |
| Psychological Distress (Interval)^a^ | 6.03  (SE = 0.12) | 6.17  (SE = 0.26) | 5.94  (SE = 0.15) | 0.700 |
| Cancer History  Yes  No | ***  267(6.1)  2030(93.9) | 113(55.5)  711(65.3) | 154(44.5)  1319(34.7) | 4.760* |
| Outcome Measurement of Contextual Factors  Excellent  Very good  Good  Fair  Very poor | ***  767(30.9)  977(41.4)  416(19.8)  103(6.3)  34(1.7) | 295(37.8)  339(32.4)  141(33.6)  37(48.3)  12(30.3) | 472(62.2)  638(67.6)  275(66.4)  66(51.7)  22(69.7) | 8.565+ |
| Consultation with Healthcare Professionals  Yes  No | ***  824(35.3)  1473(64.7) | N/A | N/A |  |
| Note:  ^a^ Psychological distress: higher scores indicate poor mental health.  †*p* ≤ 0.1.  *p ≤ 0.05.  ** *p* ≤ 0.01.  *** *p* ≤ 0.001. | | | | |
